# L’influence des prestations des médecins à la première ligne de soins sur le système intégré de district sanitaire à Kisangani, République Démocratique du Congo: une étude qualitative

**DOI:** 10.11604/pamj.2021.39.215.25737

**Published:** 2021-07-27

**Authors:** Samuel Bosongo, Faustin Chenge, Albert Mwembo, Bart Criel

**Affiliations:** 1 Centre de Connaissances en Santé au Congo, République Démocratique du Congo,; 2 Faculté de Médecine et Pharmacie, Université de Kisangani, Kisangani, République Démocratique du Congo,; 3 Ecole de Santé Publique, Université de Lubumbashi, Lubumbashi, République Démocratique du Congo,; 4 Institut de Médecine Tropicale d´Anvers, Antwerpen, Belgique

**Keywords:** Médecins, première ligne de soins, système de santé de district, République Démocratique du Congo, Kisangani, Physicians, primary care, district health system, Democratic Republic of Congo, Kisangani

## Abstract

**Introduction:**

en République Démocratique du Congo (RDC), les médecins, autrefois absents, prestent de plus en plus à la première ligne de soins, particulièrement mais pas exclusivement en milieu urbain. Cet article décrit et analyse l´influence des prestations des médecins à la première ligne sur le système de santé intégré de district à Kisangani en RDC.

**Méthodes:**

nous avons conduit 40 interviews semi-structurées au troisième trimestre 2018 auprès des acteurs du district sanitaire (population, infirmiers, médecins, gestionnaires), sélectionnés de façon raisonnée. Les questions ont porté sur la motivation des médecins, leur paquet d´activités et les perceptions des autres acteurs du district sur leurs prestations à la première ligne. Les données ont été analysées par contenu thématique.

**Résultats:**

les prestations des médecins à la première ligne est une situation de fait, non planifiée et non soutenue. Elle tire son origine en grande partie dans le besoin d´insertion professionnelle des médecins. Elle semble améliorer l´acceptabilité des soins mais en limite l´accessibilité financière. Elle est associée à un élargissement non contrôlé du paquet d´activités et crée la compétition avec la deuxième ligne.

**Conclusion:**

les prestations des médecins sont un défi et une opportunité du renforcement de la première ligne tout en préservant la complémentarité avec la deuxième. Une (re)définition du rôle et paquet d´activités des médecins à la première ligne est alors nécessaire. D´où la nécessité d´un dialogue entre les différents acteurs du système de santé pour (re)définir de manière consensuelle un modèle de première ligne de soins adapté aux médecins.

## Abstract (en)

**Introduction:**

in the DRC, doctors, formerly absent, are increasingly being employed as primary care physicians, in particular but not exclusively in urban areas. This study describes and analyses the impact of primary care physician services on the integrated district health system in Kisangani, DRC.

**Methods:**

in the third quarter of 2018, we conducted 40 semi-structured interviews of health district stakeholders (population, nurses, doctors, managers) selected in a reasoned way. Questions focused on doctors' motivation, their package of activities and the perceptions of other district stakeholders on their front-line services. Data were analysed using the thematic content analysis.

**Results:**

the services of primary care physicians were a de facto but they were unplanned and unsupported. This derived largely from doctors' need for professional integration. This seemed to improve treatment acceptability but limited their financial accessibility. It was associated with an uncontrolled expansion of the activity packages and caused competition between first-line and second-line physicians.

**Conclusion:**

physician services are a challenge and an opportunity to strengthen first-line care while preserving complementarity with second-line care. A (re)definition of first-line physicians' role and activity package is then required. Hence, the need to improve the dialogue between different health system actors in order to (re)define consensually a model of first-line care adapted to match physicians' needs.

## Introduction

Plus de quatre décennies après la déclaration d´Alma Ata en 1978 [1], les Soins de Santé Primaires (SSP) ont bénéficié d´un (re)engagement des chefs d´Etat et de gouvernement à Astana en 2018 [2]. Ils demeurent la pierre angulaire des politiques sanitaires de nombreux pays d´Afrique où le district sanitaire en est le modèle opérationnel principal. Le district sanitaire est généralement organisé en deux lignes de soins complémentaires jouant chacun un rôle spécifique: la première constituée d´un réseau pluraliste de formations sanitaires qui dispensent les soins de santé de base et la deuxième ligne formée par un (ou plusieurs) structures de référence qui offrent des soins plus techniques et spécialisés [3, 4]. En principe, la première ligne de soins sert de premier contact avec la population et offre les soins globaux (centrés sur la personne), continus, cordonnés et adaptés au besoin de chaque individu et communauté [5, 6].

Depuis la décolonisation de l´Afrique, et suite au nombre limité de médecins, l´ensemble des soins au premier échelon était délégué aux prestataires non-médecins (appelés en anglais *nurse-practitioners* ou *non-physician clinicians*) [7, 8]. Cette délégation était assortie des mesures d´encadrement (standardisation et supervision) pour assurer la qualité des soins [9]. Cependant, depuis quelques années, les médecins se sont invités dans le paysage du premier échelon de soins en Afrique. Ils y prestent selon des modèles opérationnels multiformes. La littérature fait état des modèles inspirés de la médecine de famille européenne en Afrique du Sud [10-12], au Mali, à Madagascar et au Bénin [13, 14] ou d´un modèle caractérisé par la transposition du modèle hospitalier à la première ligne de soins (PLS) en République Démocratique du Congo (RDC) [15].

En République Démocratique du Congo (RDC), ce phénomène gagne de l´ampleur surtout en milieu urbain. La proportion des formations sanitaires de première ligne (FSPL) qui emploient un médecin est passée de 68% à 81% entre 2010 et 2018 à Lubumbashi [16, 17]. Elle a été évaluée à 60% en 2018 à Kisangani [15]. Ces prestations croissantes des médecins à la première ligne ne sont pas prévues dans les normes sanitaires du pays, créant ainsi un déphasage avec la réalité [18]. Elles pourraient avoir des influences multiples sur l´organisation du système de santé de district (SSD) et son fonctionnement en tant qu´un système intégré. A notre connaissance, ces influences restent encore peu documentées et moins bien scientifiquement étudiées. L´objectif de cette étude est de décrire et analyser les influences des prestations des médecins à la PLS sur le SSD dans la ville de Kisangani en RDC.

## Méthodes

**Type et milieu d´étude:** nous avons conduit une étude de cas à visée descriptive et analytique au troisième trimestre de l´année 2018. Le cas était l´influence des prestations des médecins à la PLS sur l´intégration du SSD. L´unité d´analyse était les formations sanitaires employant les médecins dans la ville de Kisangani. La ville de Kisangani, notre milieu d´étude, est le chef-lieu de la province de la Tshopo. Elle est située au Nord-Est de la RDC. Les statistiques sanitaires indiquent que cette ville compte environ 1,2 million d´habitants répartis dans 5 districts sanitaires. Elle dispose d´une faculté de médecine qui forme en moyenne une centaine de médecins par an.

**Population d´étude et stratégies d´échantillonnage:** la population d´étude était constituée des informateurs clés choisis parmi les acteurs de différents niveaux du district sanitaire. Il s´agissait des usagers des FSPL, des prestataires non-médecins des FSPL (infirmiers principalement), des médecins prestataires dans les FSPL, des directeurs des hôpitaux généraux de référence (HGR) et des membres des équipes cadres de districts (ECD). Ils ont été sélectionnés par choix raisonné et de manière diversifiée par FSPL et graduelle jusqu´à la saturation des données.

**Figure 1:**
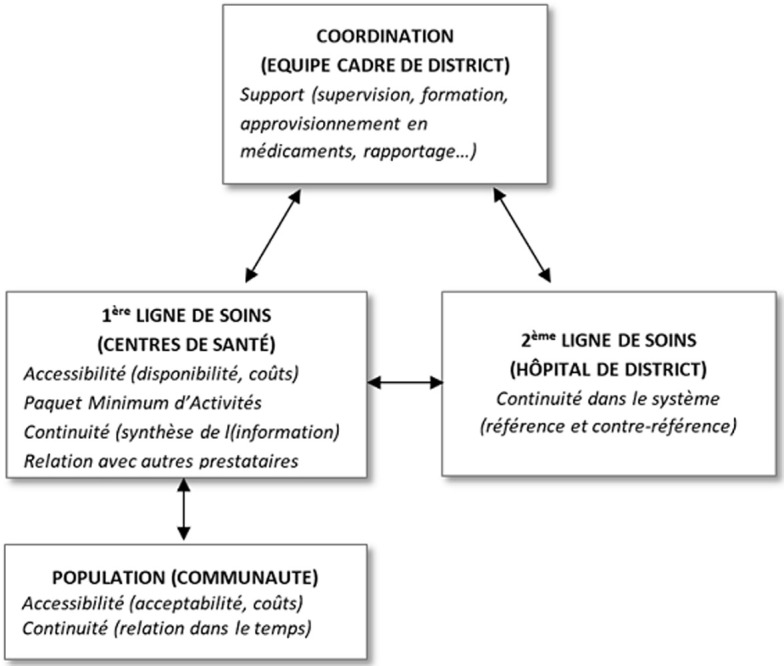
cadre conceptuel du SSD (adapté de Chenge, 2013)

**Cadre conceptuel:** nous avons considéré les principes d´organisation d´un SSD fondé sur les SSP comme cadre d´analyse. Selon Vuori [19] les SSP sont conçus sous quatre dimensions: les SSP comme (i) un ensemble d´activités, (ii) un niveau des soins, (iii) une stratégie de l´organisation des services de santé et (iv) une philosophie qui devrait imprégner l´ensemble du système de santé. Le SSD est le niveau opérationnel de mise en œuvre des SSP. Il consiste ainsi en un système de santé à deux échelons complémentaires et spécifiques placés sous le pilotage d´une structure de coordination qui est l´ECD [20]. C´est en principe un système intégré qui assure un continuum de prise en charge des problèmes de santé sans créer un chevauchement de fonctions entre les deux échelons et qui permet en son sein une circulation optimale des patients et de l´information [20, 21]. Notre analyse s´est focalisée sur l´influence des prestations des médecins, autrefois absents à la PLS sur un tel système en RDC. Plus spécifiquement, nous avons examiné cette influence sur (i) l´organisation et le fonctionnement des FSPL en termes d´accessibilité (culturelle, financière et disponibilité), de paquet de soins (curatifs, préventifs et promotionnels), de la continuité des soins dans le temps (y compris la fonction de synthèse) et de relation avec les autres professionnels de santé (travail en équipe, coordination des soins, arrangements institutionnels), (ii) la complémentarité entre les deux échelons du district traduits par la référence et contre-référence des patients entre les deux lignes de soins et (iii) la relation des médecins avec les ECD sanitaires (Figure 1).

**Collecte et analyse des données:** les données ont été collectées par des entretiens individuels semi-structurés à l´aide d´un guide d´entretien. Ces entretiens ont été conduits par le premier auteur en français pour les médecins, infirmiers, gestionnaires des HGR, membres des ECD et certains usagers de soins. Pour d´autres usagers, les entretiens ont été faits en Lingala puis traduits en Français après transcription. Les entretiens ont porté sur la motivation des médecins à prester à la PLS, le paquet d´activités des médecins à la PLS, la collaboration entre médecins et infirmiers à la PLS, les perceptions des infirmiers, des autorités sanitaires et de la population sur les prestations et implications des médecins à la PLS. Les données ont été enregistrées à l´aide d´un smartphone et par la prise des notes sur certaines attitudes des participants, et transcrits mots à mot en verbatim. L´analyse du verbatim a commencé durant la collecte des données et les résultats préliminaires de ces analyses ont permis d´approfondir les thèmes émergeants lors des interviews ultérieurs. Les données ont été analysées manuellement par contenu thématique en référence au cadre conceptuel décrit ci-haut. Nous avons procédé à la triangulation des données de différents types de participants et les résultats d´analyse ont été approuvés par les participants.

**Considérations éthiques:** le protocole de cette étude a été revu et approuvé par le comité d´éthique médicale de l´Université de Lubumbashi en date du14/06/2018, sous le numéro UNILU/CEM/092/2018. Un consentement éclairé garantissant l´anonymat et la libre participation a été obtenu par écrit avant entretien.

## Résultats

Nous avons conduit 40 interviews de 15 à 35 minutes auprès des acteurs de différents niveaux du SSD. Nous avons interviewé 10 usagers de soins, 10 infirmiers et 15 médecins au niveau des FSPL, 2 responsables des HGR et 3 membres des ECD.

**Motivation des médecins à prester à la PLS:** les médecins ont rapporté deux principales raisons qui les motivaient à prester dans les FSPL. Il s´agissait de (i) le manque d´emploi attribué à l´absence de leur recrutement dans les hôpitaux publics qui ne leur facilitait pas la survie et l´acquisition des compétences ainsi que (ii) la volonté d´aider en rapprochant les soins de la population dépourvue de moyens.

*«L´Etat congolais n´engage plus les médecins depuis un temps, alors j´ai jugé bon de travailler dans ce centre de santé pour mettre en pratique ce que j´ai appris à la faculté. Etant donné qu´Il est difficile d´acquérir les aptitudes professionnelles sans travailler. C´est en forgeant qu´on devient forgeron. La médecine en dehors d´être une science, est aussi un art. C´est en pratiquant qu´on acquiert les aptitudes.»* (MED-06).

Par ailleurs, les jeunes médecins sans emploi dans la fonction publique ont rapporté que leur présence à la PLS était temporaire et qu´ils n´y voyaient pas forcément une perspective de carrière. *«Je suis ici pour survivre et ne pas perdre ce que j´ai appris. J´espère un jour travailler dans un grand hôpital où il y a tout et pourquoi ne pas me spécialiser.»* (MED-01).

### Influence des médecins sur l´accessibilité aux soins à la PLS

Les perceptions des acteurs du SSD sur l´influence des prestations des médecins sur l´accessibilité des soins à la PLS ont pris trois orientations: l´acceptabilité, la disponibilité et l´accessibilité financière.

***Acceptabilité:*** les usagers ont rapporté la présence du médecin comme un facteur les incitants dans le choix d´utiliser une FSPL, car ils le considèrent comme un personnel sanitaire le plus compétent suite à son plus haut niveau d´étude et des savoirs.

*«Je préfère aller consulter le centre de santé avec médecin parce que les médecins ont fait des longues études, ils ont étudié le corps humain mieux que quiconque et savent quoi faire pour identifier la maladie et la traiter.» (COM-09)*.

Ces propos ont aussi été confirmés par les infirmiers des FSPL.

*«Quand les gens apprennent qu´il y a un médecin qui consulte, ils pensent qu´il va trouver solution à tous leurs problèmes de santé. Donc la présence des médecins attire les malades et augmente le nombre de consultations.»* (INF-05).

***Disponibilité:*** les usagers ont rapporté qu´il est facile de voir les médecins dans les FSPL quand ils y sont présents. Ils ont toutefois déploré le fait que les médecins prestent à plusieurs endroits et ne sont donc pas toujours permanents.

*«Nous préférons ici parce que nous sommes très vite reçus par le médecin et traités contrairement aux grands hôpitaux où on peut passer 2 à 3 jours sans être vu par un médecin. Mais les médecins ne sont pas souvent permanents ici parce qu´ils travaillent à plusieurs endroits. Et, pour les avoir il faut des moyens supplémentaires en termes de transport ou de communication.»* (COM-10).

***Accessibilité financière:*** les usagers ont rapporté que les coûts des soins des médecins étaient élevés et leur tarification ne dépendait pas du niveau de revenu du patient, frisant ainsi l´équité.

*«Leurs soins sont chers et je ne l´apprécie pas. Aussi, on n´a pas les mêmes moyens dans ce monde. Il faut tenir compte du niveau de chaque famille. Ceux qui viennent à pied ou à vélo n´ont pas le même niveau que ceux qui viennent en voiture et ne doivent pas payer la même chose.»* (COM-01).

**Influence des médecins sur le paquet des soins à la PLS:** les médecins et infirmiers des FSPL ont rapporté que le Paquet Minimum d´Activités (PMA) recommandé par le ministère de la santé n´était pas respecté. Les médecins posaient des actes hospitaliers ne relevant pas du plateau technique des FSPL. C´est notamment les cas des interventions chirurgicales pour lesquelles les médecins avancent les raisons suivantes de leur réalisation dans les FSPL: (i) la valorisation professionnelle des médecins, estimant que le PMA en vigueur n´était pas adapté à leur niveau; (ii) la survie institutionnelle car les activités du paquet minimum étaient peu rentables pour supporter les coûts de fonctionnement; et (iii) la demande pressante des patients qui exigeaient des soins hospitaliers à la PLS. Ils refusaient les références dans les HGR pour diverses raisons notamment la méconnaissance du rôle de l´HGR et de la nécessité de référence, le coût élevé des soins et le mauvais accueil.

*«Je fais tout ce qu´un médecin peut faire, de la consultation aux actes chirurgicaux. […] Je suis conscient que ce n´est pas du PMA, mais je pense aussi que ce PMA n´était pas conçu pour nous médecins. Faut-il vraiment référer un patient à l´hôpital général pour un acte qu´on peut poser ici tout simplement parce que ce n´est pas dans le PMA? Soyons réalistes!»* (MED-04).

*«A l´hôpital, il y a des médecins; ici aussi, il y a un médecin; ce que les médecins de là peuvent faire, le médecin d´ici peut aussi le faire. Alors pourquoi nous envoyer là où les soins coûtent chers, où on ne s´occupe pas bien de nous? Nous préférons rester ici.»* (COM-07).

### Influence des médecins sur la continuité des soins

***Continuité dans le temps et fonction de synthèse:*** certains usagers ont rapporté leur souhait d´être vus par un même médecin sur une longue durée. Mais les changements fréquents des médecins dans les FSPL ne les permettent pas. Les médecins et les infirmiers ont reconnu l´absence des dossiers médicaux comme support à la fonction de synthèse de la PLS. Cette situation concernait la majorité des FSPL indépendamment de la présence des médecins.

*«Pour chaque patient, on établit une fiche de consultation et à la fin de l´épisode de la maladie, on classe la fiche. Si le même patient se présente encore pour un autre épisode, on établit une autre fiche et ainsi de suite. Les fiches sont assemblées à la fin de chaque mois et classées dans les archives, ce n´est pas facile de les retrouver à chaque consultation.»* (INF-02).

***Continuité dans le système:*** les responsables des HGR ont rapporté que la présence des médecins dans les FSPL créait une compétition avec leurs hôpitaux. Selon eux, l´insuffisance de la régulation en était la cause.

*«Ils font tout là-bas, je dis bien tout, ils opèrent toute sorte de maladie, ils hospitalisent toute sorte de patient, et on ne sait plus faire la différence entre un hôpital et un centre de santé. C´est un peu comme dans la jungle, si je peux dire ainsi (rire). Ils réfèrent seulement des malades pauvres après les avoir tout prix et des malades agonisants pour éviter qu´ils meurent chez eux.»* (HGR-01).

Les membres des ECD ont rapporté que cette compétition impactait négativement sur les indicateurs des hôpitaux et les performances des districts. Ils ont aussi reconnu leur impuissance dans la régulation des prestations des médecins à la PLS.

*«Qu´est-ce qu´on peut faire à notre niveau si l´Etat lui-même n´arrive pas à maîtriser la situation. Il y a à boire et à manger dans cette situation, je vous le dis»* (ECD-01).

**Relations avec les autres prestataires de la PLS:** les médecins non-propriétaires des FSPL ont rapporté n´avoir aucun rôle administratif dans les FSPL où ils œuvrent. Ceci n´était pas le cas des médecins propriétaires des FSPL qui avaient un pouvoir administratif sur les autres prestataires.

*«Je me considère comme un visiteur, je passe deux à trois fois par semaine pour mes prestations cliniques. Je ne m´intéresse presque pas aux aspects administratifs car c´est la charge de l´infirmier titulaire.»* (MED-03).

Les médecins et les infirmiers ont rapporté que leur relation était plus technique et caractérisée par la complémentarité et la collaboration.

*«La prise en charge des patients demande qu´il y ait une bonne collaboration au sein de l´équipe. Le médecin n´est pas tout à fait un seigneur ou quelqu´un qui connait tout ou qui s´auto-suffit. Nous essayons de collaborer avec les infirmiers pour essayer d´améliorer la qualité de la prise en charge de nos patients. Le médecin prescrit le traitement et l´infirmier exécute les prescriptions du médecin.»* (MED-02).

Selon les infirmiers, cette collaboration était parfois aussi émaillée de conflits suite notamment au manque de considération et de réceptivité envers les collaborateurs, noté chez certains médecins.

*«Ils commettent parfois des erreurs mais n´aiment pas qu´on leur reproche. C´est vraiment dommage que certains croient tout savoir et ne considèrent pas nos points de vue. Ils perçoivent nos remarques comme une humiliation ou une remise en cause de leurs diplômes.»* (INF-01).

**Relations des médecins avec les membres de l´ECD:** les médecins non-propriétaires des FSPL ont rapporté qu´ils ne bénéficiaient d´aucun support des ECD tandis que les médecins propriétaires ont rapporté une bonne collaboration avec elles.

*«Je ne collabore pas directement avec eux parce qu´ils ne me reconnaissent pas et ne m´impliquent pas dans leurs activités comme les formations, campagnes de vaccination et autres. C´est avec l´infirmier titulaire qu´ils collaborent.»* (MED-09).

Les membres des ECD ont fustigé les prestations «clandestines» des médecins à la PLS, ce qui ne facilite pas leur encadrement. Ils ont suggéré une concertation préalable pour l´harmonisation des attentes entre les ECD et les propriétaires des FSPL qui emploient les médecins.

*«La majorité des médecins qui prestent dans les centres de santé le font de leur propre gré ou sur base des attentes privées avec les propriétaires de ces centres sans que nous ne soyons informés. Dans ces conditions, comment pouvons-nous nous intéresser aux personnes que nous ne connaissons pas? Comment pouvons-nous les encadrer?»* (ECD-01).

## Discussion

Cette étude a montré que les prestations des médecins à la PLS ne sont pas sans influence sur le SSD dans la ville de Kisangani. C´est une situation de fait en RDC, non planifiée et non soutenue jusqu´à ce jour. Elle tire son origine en grande partie dans le besoin d´insertion professionnelle des médecins. Elle semble influencer positivement l´acceptabilité des soins à la PLS mais en limiter l´accessibilité financière et l´équité. Elle est associée à un élargissement du PMA, mais crée la compétition entre les deux échelons des soins du district.

**Une situation de fait, non planifiée, non soutenue et non voulue:** le fait que la présence des médecins à la PLS tire son origine en grande partie dans le besoin de l´insertion professionnelle des médecins, tel que trouvé dans cette étude, a été aussi trouvé par d´autres études réalisées au Mali, à Madagascar et au Bénin [14, 22, 23]. Cependant, deux différences majeures ont été notées entre la situation de ces pays et celle vécue en RDC. La première se situe au niveau du contexte et la seconde au niveau de la planification et du soutien institutionnel. En effet, la pratique des médecins à la PLS dans ces pays concernait les zones rurales. En RDC cependant, elle concerne surtout le milieu urbain n´ayant pas forcément les mêmes caractéristiques [16-18]. Aussi, dans ces pays, cette pratique a bénéficié d´une institutionnalisation (partielle soit-elle) et d´un accompagnement professionnel [14, 22, 23]. Il en était aussi le cas du développement de la médecine de famille en Afrique du Sud [12] et au Cuba [7]. En RDC cependant, il s´agit d´une situation de fait qui ne trouve pas sa place dans les normes sanitaires du pays. C´est ce qui expliquerait l´absence de son accompagnement par les autorités sanitaires. C´est aussi une situation non voulue même par les médecins concernés qui la considèrent comme une «situation professionnelle transitoire». Cet engagement inconstant a été également observé auprès des médecins généralistes communautaires du Bénin et du Mali, en dépit de l´accompagnement dont ils bénéficiaient [22, 23].

**Une plus-value pour l´acceptabilité, mais une limite à l´accessibilité financière:** nos résultats ont montré que les prestations des médecins à la PLS semblent améliorer l´acceptabilité des soins par la population. Ils vont dans le même sens que ceux de Manzambi *et al*. [24] à Kinshasa (RDC) et de Codjia *et al*. [22] au Mali. Bien que les prestations des médecins paraissent une plus-value pour l´acceptabilité des soins à la PLS, elles constituent aussi une limite à l´accessibilité financière. Cette limite a été aussi trouvée par Codjia *et al*. [22] au Mali, où la présence des médecins dans les structures sanitaires générait une augmentation des coûts et influençait les variations des tarifs pratiqués.

**Une opportunité d´élargir le paquet de soins, mais une menace à l´intégration du SSD:** nous avons observé que la prestation des médecins à la PLS était associée à l´élargissement du PMA. Cette observation a été également faite au Mali, à Madagascar et au Bénin [14, 22, 23] où les médecins élargissaient le registre des problèmes de santé traités et permettaient la prise en charge spécialisée de quelques pathologies aux centres de santé. C´est aussi ce que Kaya *et al*. [17] ont observé à Lubumbashi (en RDC) où 82% des médecins étaient impliqués dans la prise en charge de l´hypertension et du diabète dans les FSPL. Cependant, dans un environnement peu ou pas régulé, cet élargissement du PMA, motivée en partie par la survie institutionnelle, est susceptible d´occasionner la marchandisation des soins et en limiter l´accès financier et la qualité technique. En outre, l´élargissement non régulé du PMA observé dans cette étude crée une compétition entre les deux lignes de soins du district. Ainsi, il met à mal les principes de spécificité et de complémentarité du SSD intégré [20, 21]. Ce résultat corrobore celui de Bosongo *et al*. [18] à Kisangani où la transposition du modèle hospitalier à la PLS était le mode opérationnel dominant de la pratique des médecins à la PLS. Les faibles taux de référence (1,2%) et de contre-référence (32%), la forte utilisation des hôpitaux par les malades non référés en consultations externes (92%) et le faible taux moyen d´occupation des lits des hôpitaux (37,5%) rapportés dans le DHIS2 (District Health Information System, version 2) par les cinq districts sanitaires de la ville de Kisangani au premier semestre 2018 reflètent bien cette compétition. Desplats *et al*. [8] ont même parlé des FSPL *«petits hôpitaux»* et des hôpitaux *«gros dispensaires»*.

**Implications pour la pratique:** les prestations des médecins peuvent être considérées à la fois comme une opportunité et défis du renforcement de la PLS tout en préservant la complémentarité entre les deux lignes de soins. Elles nécessitent un vrai dialogue entre les différents acteurs du système de santé pour s´accorder sur un modèle de PLS plus adapté aux médecins sans empiéter les fonctions de l´hôpital. D´où la nécessité de réunir les conditions optimales de prestation des médecins à la PLS définies par Bosongo [18]: volonté politique, révision des normes d´organisation et de fonctionnement de la PLS en termes de ressources humaines, d´infrastructures, d´équipements, des médicaments essentiels, de paquet d´activités et de support de l´ECD. Agir ainsi serait, à notre avis, une forme de régulation par encouragement selon Montagu et Goodman [25].

**Limites de l´étude:** cette étude s´est limitée à recueillir les données au niveau des acteurs de différentes composantes du district sanitaire. De ce fait, elle ne présente pas une image complète de la réalité dans la mesure où les perceptions des acteurs du niveau provincial ou national n´ont pas été recueillies. Ceux-ci ont, en principe, un rôle important à jouer dans la régulation des soins, notée insuffisante sur terrain. L´acceptabilité des soins étant un reflet de la qualité (des soins) perçue par la population, cette étude n´a pas approfondi les composantes de la qualité des soins. Il y a dès lors une nécessité d´étudier l´éventuelle plus-value des prestations des médecins à la PLS en termes de qualité des soins.

## Conclusion

Les médecins se positionnent de plus en plus comme des acteurs majeurs de la PLS en RDC, surtout en milieu. Ce positionnement n´est pas sans conséquences sur le SSD en tant que système intégré. En effet, les prestations des médecins à la PLS semblent améliorer l´acceptabilité des soins par la population et élargissent le paquet de soins offert à ce niveau. Elles limitent l´accessibilité financière (et l´équité) aux soins et ruine la complémentarité entre les deux lignes de soins du district sanitaire comme elle ne bénéficie pas d´un encadrement institutionnel (régulation). Il y a donc une nécessité d´un dialogue entre les acteurs du système de santé à différents niveaux pour (re)définir de manière consensuelle un modèle de PLS adapté aux médecins.

### Etat des connaissances sur le sujet

- La prestation des médecins à la PLS est un phénomène émergent et croissant en Afrique et tire en grande partie son origine dans le besoin de l´insertion professionnelle des médecins;
- Les modèles opérationnels de prestation des médecins à la PLS sont multiformes et inspirés en partie par la médecine de famille.

### Contribution de notre étude à la connaissance

- L´élargissement non régulé du paquet de soins à PLS crée une compétition entre les deux échelons du district sanitaire et met à mal les principes de spécificité et de complémentarité du SSD intégré;
- Si elle est encadrée, la présence des médecins peut constituer une opportunité de renforcement de PLS tout en préservant la complémentarité entre les deux échelons de soins.

## Remerciements

Nous remercions tous ceux qui nous ont facilité la réalisation de ce travail : Drs Geneviève Michaux, Kéfilath Bello, Samuel Makali, Charles Kaya pour les contributions à l´élaboration du protocole de cette étude ; Dr Didier Chuy pour sa relecture critique de la version finale du manuscrit ; nos informateurs clés pour leur participation et validation des résultats de cette étude et les autorités sanitaires de la province de la Tshopo pour avoir autorisé la réalisation de cette étude.

## References

[1] (1978). Declaration of Alma-Ata International Conference on Primary Health Care, Alma-Ata, USSR.

[2] Déclaration d´Astana (2018). Global conference on primary health care. Astana.

[3] Criel B, Garcia M, Pirard M (2016). Organisation des services de santé Institut de Médecine Tropicale. Anvers.

[4] World Health Organization (1987). Division of Strengthening of Health. Declaration on strengthening district health systems based on primary health care.

[5] World Health Organization (WHO) (2008). Primary Health Care: Now More Than Ever.

[6] Expert Panel on Effective Ways of Investing in Health (2014). Definition of a frame of reference in relation to primary care with a special emphasis on financing systems and referral systems.

[7] Dugas S, Van Dormael M (2003). La construction de la médecine de famille dans les pays en développement. Studies in Health Services Organisation & Policy.

[8] Desplats D, Koné Y, Razakarison C (2004). Pour une médecine générale communautaire en première ligne. Médecine Trop.

[9] Equipe du Projet Kasongo (1976). Utilisation du personnel auxiliaire dans les services de sante ruraux: Une experience au Zaire. Bull World Health Organ.

[10] Pasio KS, Mash R, Naledi T (2014). Development of a family physician impact assessment tool in the district health system of the Western Cape Province, South Africa. BMC Fam Pract.

[11] Swanepoel M, Mash B, Naledi T (2014). Assessment of the impact of family physicians in the district health system of the Western Cape, South Africa. African J Prim Heal Care Fam Med.

[12] Mash B (2011). Reflections on the development of family medicine in the Western Cape: A 15-year review. South African Fam Pract.

[13] Desplats D (2013). The feasibility of community-based private medical practice in Africa and Madagascar. Facts Rep.

[14] Caplain R, Yacoubou I, Adedemy D, Sani A, Takam S, Desplats D (2014). Promouvoir des soins de proximité en Afrique: l´exemple de la médecine générale communautaire au Bénin. Santé Publique.

[15] Bosongo S, Chenge F, Mwembo A, Criel B (2617). Les médecins prestataires à la première ligne des soins dans la ville de Kisangani en République Démocratique du Congo: vers une typologie. Manuscrit soumis à la revue African Journal of Primary Health & Family Medicine sous le numéro ID.

[16] Chenge M, Van der Vennet J, Porignon D, Luboya N, Kabyla I, Criel B (2010). La carte sanitaire de la ville de Lubumbashi, République Démocratique du Congo Partie I: problématique de la couverture sanitaire en milieu urbain congolais. Glob Health Promot.

[17] Kaya M, Chuy K, Chenge M, Mwembo T, Mashini N, Bisimwa B (2020). Prestations des médecins au niveau de la première ligne des soins dans la ville de Lubumbashi, République Démocratique du Congo. IOSR J Nurs Heal Sci.

[18] Bosongo S (2017). La médicalisation de la première ligne de soins en milieu urbain: Quel modèle pour un système de santé fort en République Démocratique du Congo? Cas de la ville de Kisangani. Mémoire de master. Institut de Médecine Tropicale d´Anvers.

[19] Vuori H (1986). Health for all, primary health care and general practitioners. J R Coll Gen Pract.

[20] Chenge F (2013). De la nécessité d´adapter le modèle de district au contexte urbain: Exemple de la ville de Lubumbashi en RD Congo. Studies in Health Services Organisation & Policy.

[21] Van Lerberghe W, Lafort Y (1990). Rôle de l´hôpital dans le district: Dispenser ou soutenir les soins de santé primaires?. Division de renforcement des services de la santé, Organisation Mondiale de la Santé.

[22] Codjia L, Jabot F, Dubois H (2010). Accroitre l´ accès aux personnels de santé dans les zones rurales ou reculées. Etude de cas 2 Evaluation du programme d´appui à la médicalisation des aires de santé rurale au Mali. OMS.

[23] Guévart E (2015). Evaluation du projet: «Médicalisation des zones rurales défavorisées du Nord Bénin» (2009-2012) et «Renforcement et extension du programme d´installation de médecins généralistes communautaires (MGC) en zones rurales dans le nord Bénin» (2012-2014).

[24] Manzambi JK, Tellier V, Bertrand F, Albert A, Reginster JY, Balen H (2000). Les déterminants du comportement de recours au centre de santé en milieu urbain africain: résultats d´une enquête de ménage menée à Kinshasa, Congo. Trop Med Int Heal.

[25] Montagu D, Goodman C (2016). Prohibit, constrain, encourage, or purchase: how should we engage with the private health-care sector?. Lancet.

